# Public or Private Schools

**Published:** 1921-03-05

**Authors:** 


### THE PUBLIC WELL-BEING.

### Public or Private Schools.

The greatest industry of this country is education. Compared with making boots or books or butter, making men and women is of infinite importance. Men and women are the end of all industry and the raw material of every industry. The medical profession seized this practical fact long before the community in general did so, and the presence for many years in every school of doctor and nurse means that they realise that the most vital part of the empire is the school. Its worst vices can be checked there. Its virtues can there be developed. The teacher and the doctor are rapidly drawing closer together, and the day is not far distant when it will be generally acknowledged that they cannot do their work apart, and that neither can be effective with the co-operation of the other. What interests one interests both, and the discussion which has cropped up once again on the question of the public versus the private school is one in which everybody who is interested in the type of manhood we are to produce is bound to have something to say. It is an interesting sidelight on the many layers of which our history is made up that the public school of to-day should be the really private school, having carried its name over from the middle ages, when religion was one of the good works of the monks or the enterprise of some pious benefactor or founder who would have been staggered if he could have seen the years through which his benefaction would last and the extent to which by the ordinary increase of wealth it would grow. The new Education Act, which has laid down a broad highway practically from the cradle to the professor's chair, raises the question of the place of these private institutions in the future. They are at present independent establishments, more independent of their neighbours, though perhaps a little less jealous of each other, than the Greek city states.

Two things have minimised the effects of this complete independence. In the first place, the great public schools have entered so thoroughly into the spirit of the nation, produced its men of affairs and influence, and identified themselves to such an extent with the national tradition that their practical independence has almost been lost sight of. The meaning of this fact will come home to the reader if he will try to imagine these schools imbued with a tradition consistently opposed to that of the nation as a whole—extreme communism, say. The second factor which has brought the public schools into harmony is that their eyes have always been turned to Oxford or Cambridge; and the need for passing on their best scholars to one of the older universities has led to an extremely large measure of conformity. The existence of the new universities with their different traditions and their insistence on modern subjects and their comparative carelessness of such previous essentials as Latin and Greek have done much to break down this uniformity.

The public schools have represented what is considered the very type of British manhood, and yet they are being challenged. One reason is the spread of the spirit which lies behind the new Education Act. Education now is a right like the vote. Men believe they owe it to every child irrespective of his parentage or his future. The growth of machinery in peace and war has narrowed the gulf between leaders and led which made the public-school boy so valuable as an officer in the services or a leader in pioneering. The condition of the world and the demands upon Englishmen are changing, and the mechanic, the oil stoker of a great liner, the cowboy who can handle a motor tractor and keep accounts, have taken the place of the men who could use their muscles if their leader could use his brains. The one supreme gift of the English public school, however, is not technically educational at all; and it is a gift which has been lavished on the world. Law in India, liberty in America, and order in most continents owe much to this best quality of the English public school. It is the quality we call sportsmanship. It is not at all the faculty to play these games which are the ridicule and the envy of the world. It is the spirit they breed of fair play and recognition of opponents' rights. This is the thing that for an empire like ours it is vital to retain. It is being fostered more than ever in our real public schools to an extent not commonly realised by those outside. The fact that it is so is evidence of the growing freedom which is being allowed in these State and municipal schools. What contestants for one and another overlook is the extent to which they have drawn together in this representative point and in many others. The way in which they are actually approximating and actually mixing in type suggests that the question of their rivalry may eventually be solved on unlooked-for lines.

